# APOBEC3G Is a p53-Dependent Restriction Factor in Respiratory Syncytial Virus Infection of Human Cells Included in the p53/Immune Axis

**DOI:** 10.3390/ijms242316793

**Published:** 2023-11-27

**Authors:** Wesley Gladwell, Oriana Yost, Heather Li, Whitney J. Bell, Shih-Heng Chen, James M. Ward, Steven R. Kleeberger, Michael A. Resnick, Daniel Menendez

**Affiliations:** 1 Immunity, Inflammation, and Disease Laboratory, National Institute of Environmental Health Sciences, National Institute of Health, Research Triangle Park, Durham, NC 27709, USA; gladwell@niehs.nih.gov (W.G.); oriana.yost@outlook.com (O.Y.); hyilianl25@gmail.com (H.L.); whitneyjbell@gmail.com (W.J.B.); kleebergers@bellsouth.net (S.R.K.); 2 Genome Integrity and Structural Biology Laboratory, National Institute of Environmental Health Sciences, National Institute of Health, Research Triangle Park, Durham, NC 27709, USA; 3 Viral Vector Core Facility, National Institute of Environmental Health Sciences, National Institute of Health, Research Triangle Park, Durham, NC 27709, USA; chens3@niehs.nih.gov; 4 Integrative Bioinformatics Support Group, National Institute of Environmental Health Sciences, National Institute of Health, Research Triangle Park, Durham, NC 27709, USA

**Keywords:** APOBEC3G, gene expression, immune response, restriction factor, p53, RSV

## Abstract

Identifying and understanding genetic factors that influence the propagation of the human respiratory syncytial virus (RSV) can lead to health benefits and possibly augment recent vaccine approaches. We previously identified a p53/immune axis in which the tumor suppressor p53 directly regulates the expression of immune system genes, including the seven members of the APOBEC3 family of DNA cytidine deaminases (A3), which are innate immune sentinels against viral infections. Here, we examined the potential p53 and A3 influence in RSV infection, as well as the overall p53-dependent cellular and p53/immune axis responses to infection. Using a paired p53 model system of p53+ and p53- human lung tumor cells, we found that RSV infection activates p53, leading to the altered p53-dependent expression of *A3D*, *A3F*, and *A3G,* along with p53 site-specific binding. Focusing on A3G because of its 10-fold-greater p53 responsiveness to RSV, the overexpression of *A3G* can reduce RSV viral replication and syncytial formation. We also observed that RSV-infected cells undergo p53-dependent apoptosis. The study was expanded to globally address at the transcriptional level the p53/immune axis response to RSV. Nearly 100 genes can be directly targeted by the p53/immune axis during RSV infection based on our p53BAER analysis (Binding And Expression Resource). Overall, we identify A3G as a potential p53-responsive restriction factor in RSV infection. These findings have significant implications for RSV clinical and therapeutic studies and other p53-influenced viral infections, including using p53 adjuvants to boost the response of *A3* genes.

## 1. Introduction

The human respiratory syncytial virus (RSV) is an enveloped, non-segmented, single-stranded RNA virus belonging to the *Paramyxoviridae* family. RSV infection is the primary cause of respiratory problems such as pneumonia and bronchiolitis in infants and young children, resulting in a significant amount of morbidity and mortality worldwide [1]. Almost all children are infected by RSV at least once before the age of two [2]. RSV also poses a significant respiratory disease burden in elderly and immunocompromised individuals [3]. Since individuals do not develop long-lasting immunity to RSV, they can become infected repeatedly throughout their lifetime. Currently, several clinical trials are being conducted to identify RSV vaccine candidates [4]. Recently, two protein-based vaccines, Arexvy (GlaxoSmithKline Biologicals) and Abrysvo (Pfizer, New York, NY, USA), have been approved for use. Arexvy is intended for individuals aged 60 and above, while Abrysvo is approved for infants up to 6 months and individuals aged 60 and above [5,6].

Cellular stress responses can protect against many endogenous and exogenous challenges, including viral infection. In mammals, the tumor suppressor p53 is a primary mediator of many stress responses [7]. Activated p53 functions mainly as a transcription factor that can regulate the expression of gene products to promote permanent cell cycle withdrawal, senescence, or apoptotic cell death, all of which result in eliminating dam-aged cells that may threaten an organism [8].

Virus infections are known to activate or interact with the p53 pathway. p53 is in-volved in the life cycle of numerous types of viruses, including the adenovirus, SV40, papillomavirus, Epstein–Barr virus, poxviruses, Zika virus, West Nile virus, influenza, human immunodeficiency virus type 1, human herpes simplex virus 1, and human coronavirus (reviewed in [9,10]). Several viral families have evolved countermeasures to inactivate the p53 pathway, emphasizing the importance of p53 during the host immune response to viral infections [11].

A broad direct connection exists between p53 and the expression of immunity-related genes referred to as the p53/immune axis. Several studies have identified p53 target genes involved in inflammation, pathogen sensing, cytokine/chemokine/interferon production, immune checkpoint regulation, and immune cell activation [10,12,13,14]. Although the primary outcome of p53 induction by viruses is p53-dependent apoptosis, p53 also regulates the expression of antiviral response components such as *IRF5*, *IRF9*, *PKR*, *TLR3*, and *ISG15*. Furthermore, IFN-α/β signaling triggers p53 transcription, creating a positive feedback loop that enhances antiviral immune responses [15]. Thus, p53 is a central mediator and amplifier of global innate immune responses. This led us [13], and, subsequently, Muñoz-Fontela et al., [14] to propose another guardian function for p53—guardian of immune integrity.

We previously demonstrated that, in response to stress signals, p53 could directly affect the expression of innate immune members of the toll-like receptor (*TLR*) and apolipo-protein B mRNA-editing enzyme, catalytic subunit 3 (*APOBEC3*) gene families [16,17,18]. The seven members of the *APOBEC3* gene family (*A3*; *A3A* to *A3H*) encode nucleic acid cytidine deaminases. The A3s catalyze C-to-U deamination of foreign single-stranded DNA or RNA, resulting in hypermutation due to C to T (or U) transitions in the genome. Most A3 subfamily members change cytosines in a 5′TC dinucleotide context, while A3G favors the deamination of the second cytosine of 5′-CC dinucleotide motifs [19,20]. As interferon-responsive genes, A3s are considered sentinels in the innate host defense immune response [21]. They can also block the replication of various viruses (reviewed in [22,23] and references therein), including retroviruses, double-stranded DNA viruses, single-stranded DNA and single-stranded (ss) RNA viruses such as the human coronavirus NL63, and the measles virus. Notably, A3G demonstrates strong antiviral activity against retrotransposons and retroviruses such as immunodeficiency virus type 1 (HIV-1) [24]. In infected cells, A3G extensively changes cytosine residues in the viral genome by deaminating them during reverse transcription. This causes massive hypermutation of G-to-A in the new viral DNA genome, making either the reverse transcripts unstable or producing defective viral proteins [25,26].

Previous studies examining the effects of p53 on RSV replication have yielded inconclusive results. While some studies suggest that RSV causes the downregulation of p53 and apoptosis, others indicate the opposite, where RSV induces p53 and its downstream pathway [27,28,29,30]. Informed by rules established in studies with p53 model cancer lines, we reported a link between RSV infection and p53 through the regulation of TLR8 that involves a p53-responsive SNP response element [31] and suggested that knowledge of allelic differences could guide the diagnosis and prognosis of RSV disease.

We hypothesize that the p53/A3 axis plays a role in human cell immune responses to RSV infection. Here, we have used a paired p53+ and p53- model system of lung cancer cells (A549 and H1299, respectively) to study this role, as well as the relationship between p53 activation and global immune responses at the transcriptional and cellular levels during RSV infection.

## 2. Results

### 2.1. Influence of p53 on the Expression of APOBEC3 Genes during RSV Infection in Human Lung Cancer Cells

**Figure 1:**
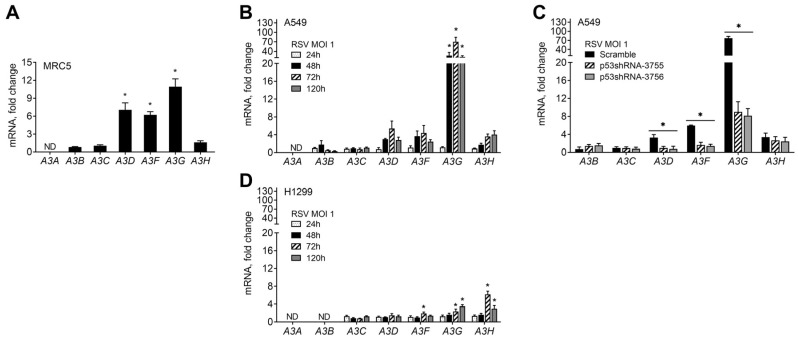
RSV-infection-induced expression of *A3* genes in human lung cells. (**A**) RSV infection induces *A3* family gene expression in normal human lung fibroblasts MRC5 cells infected with RSV at MOI of 1 for 72 h determined by qPCR. Expression changes are presented as fold-change relative to mock (PBS) non-infected cells. * *p* < 0.05 compared with non-infected cells. (**B**) Time course of induced expression of *A3* genes in A549 cells infected with RSV at MOI of 1. * *p* < 0.05. (**C**) Expression of *A3* genes in A549 cells stably expressing scrambled shRNA or p53 shRNAi vectors (p53sh-3755 or p53sh-3756) and infected for 72 h with RSV at MOI of 1. Values are displayed as fold-changes relative to their respective parental mock non-infected A549 cells. * *p* < 0.05 compared to scramble cells. (**D**) Time course of induced expression of *A3* genes in H1299 cells infected with RSV at MOI of 1. * *p* < 0.05 compared with non-infected cells.

We first asked if RSV infection could influence the *A3* gene family expression within MRC5 human lung primary fibroblasts, which is wild-type for the TP53 gene. A 72 h infection by the RSV-A2 strain using a multiplicity of infection (MOI) of 1 led to significant increases of >6-fold in *A3D* and *A3F*, and >10-fold in *A3G* (Figure 1A). No significant changes were observed for *A3B*, *A3C*, and *A3H*, while *A3A* expression was not detected. Given our earlier findings that stress-activated p53 can regulate the expression of several members of the *A3* gene family [17], we investigated the potential role of p53 in the expression of *A3* genes during RSV infection by utilizing a well-defined model system of human lung adenocarcinoma cells that included paired p53+ A549 and p53- H1299 cell lines. This system has proven useful for investigating the potential impacts of activated p53 on its diverse biological outcomes, particularly in characterizing the potential for p53 to directly influence gene target expression in response to various stresses [17,32,33,34,35].

We infected A549 cells with RSV at MOIs of 0.5 and 1 and monitored the expressions of the seven *A3* genes from 24 h to 120 h post infection (p.i.). Of the seven *A3* genes, only *A3A* was not expressed. Compared to uninfected cells, we found that RSV infections at an MOI of 1 caused the increased expression of *A3D*, *A3F*, *A3G*, and *A3H*. The expression of *A3D*, *A3F*, and *A3G* increased as early as 48 h after infection, while *A3H* was detected at 72 h p.i. (Figure 1B). As shown in Figure S1A, a similar *A3* expression profile was observed at the lower MOI of 0.5, where the expression of *A3D* and *A3G* increased as early as 48 h after infection; *A3F* and *A3H* were detected at 72 h p.i. The highest inducibility for the *A3* genes occurred 72 h after infection, with the most significant relative increase found for *A3G* at both MOIs. At 120 h after infection, the expression of all *A3*-induced genes was diminished but still significantly higher than in uninfected cells. *A3C* levels did not change, while *A3B* showed an irregular, mostly repressed expression profile, except for a slight induction at 48 h p.i. at an MOI of 1. We confirmed that RSV-induced changes in *A3* gene expression were due to p53 using A549 cells that stably express p53-shRNAi [16,36]. Following RSV exposure, the upregulation of *A3D*, *A3F*, and *A3G* was reduced or lost entirely in the p53-shRNAi cells at both the 0.5 and 1 MOIs. Since the RSV-induced *A3H* expression was unaffected, the expression of this gene was confirmed to be p53-independent (Figure 1C and Figure S1B).

To further investigate the potential role of p53 in the expression of *A3s* during RSV infection, we analyzed the *A3* expression profile in p53-null H1299 cells. Interestingly, for both MOIs, we observed only a modest induction of the *A3* genes and only a slight increase in the expression of *A3F*, *A3G*, and *A3H* after 72 h p.i., regardless of the virus amount used to infect the cells (Figure 1D and Figure S1B). The expression of *A3A* and *A3B* was not detected in H1299. There were significant differences in the *A3* gene expression profiles between p53+ and p53- cells in response to viral infection, even though their basal gene expression levels were similar (Figure 1B,D and Table S1). We investigated whether the differences in *A3* expression could be due to differences in infection levels. A recombinant-GFP-expressing RSV A2 strain (rg-RSV) was used to monitor the infection with flow cytometry. The results in Figure S1D show that both cell lines had similar numbers of RSV-GFP-infected cells after 72 h and 120 h p.i. We also utilized ddPCR to examine the RSV-*F* and -*N* gene expression. At an MOI of 1, both genes showed an increased expression in A549 and H1299 cells over time (Figure S1E,F). While the RSV-*F* gene expression was slightly lower in H1299, no RSV-*N* gene expression differences existed between the two cell lines. Thus, both cell lines have similar RSV infection rates.

**Figure 2:**
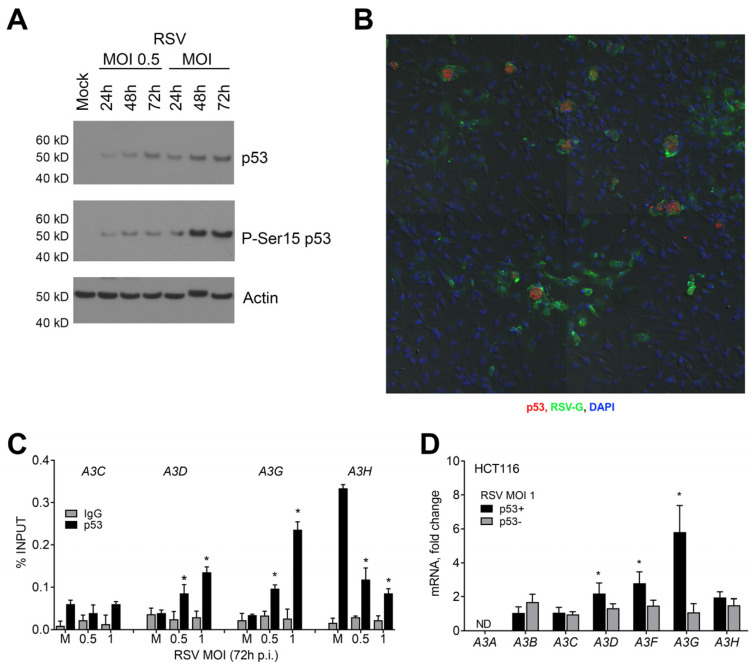
Activated p53 enhances RSV-induced *A3* gene family expression. (**A**) RSV induces p53 protein in A549 cells. Presented is a representative immunoblot against total p53 and p53 Ser15 phosphorylation. Actin detection was used as a loading control. (**B**) Representative microscopy confocal microscopy shows co-localization of p53 in RSV-infected A549 cells with MOI of 1 after 72 h p.i. (20×). RSV-G protein: FITC (green); p53: Alexa648 (red); nuclei: DAPI (blue). (**C**) RSV-activated p53 binds transcriptional regulatory regions of *A3* genes in A549 cells. p53 occupancy, determined by ChIP-PCR at 72 h p.i., is presented as the percentage of total input DNA. * *p* < 0.05 when compared with non-infected mock (M) samples. (**D**) RSV infection induces *A3* family gene expression in HCT116 colon cancer cells in a p53-dependent manner. HCT116 p53+ and p53- cells were infected with RSV at MOI of 1 for 72 h. * *p* < 0.05 compared with mock non-infected cells.

Consistent with the role of p53 in regulating the *A3* expression during RSV infection, there was substantial p53 activation/stabilization, as shown by p53 phosphorylation at serine 15 (Figure 2A). Furthermore, the p53 protein co-localized with RSV-infected cells (Figure 2B). Figure 2C shows a dose-dependent change in p53 occupancy in the promoter regions of *A3D* and *A3G* genes 72 h after infection. These regions contain a p53-response element (p53RE) [17]. However, p53 occupancy levels did not change for the p53RE in the *A3C* gene, whose expression remained unchanged after infection. For *A3H*, whose expression was p53-independent, the p53 bound levels at its p53RE decreased significantly after infection despite the high p53 occupancy observed in mock-treated cells. (*A3F* occupancy was not addressable because of sequence constraints on creating efficient primer pairs.) Our findings suggest that the p53 binding and subsequent induced expression of *A3* genes are selective during RSV infection.

In order to investigate the impact of RSV infection on the relationship between p53 and *A3* expression, we conducted experiments on isogenic colon cancer cell lines HCT116 p53+ and HCT116 p53- [37]. Our results showed that, at 72 h p.i., there were changes in the *A3D*, *A3F*, and *A3G* expression only in p53+ cells, indicating p53 involvement (Figure 2D). A3H expression, on the other hand, remained unchanged in both cell lines. These findings suggest that RSV infection can significantly increase the expression of *A3D*, *A3F*, and *A3G* genes by activating p53 in both lung and colon cancer cells. Overall, our study demonstrates a general impact of RSV on the p53-determined *A3* expression relationship.

### 2.2. Effect of A3G on RSV Infectivity

**Figure 3:**
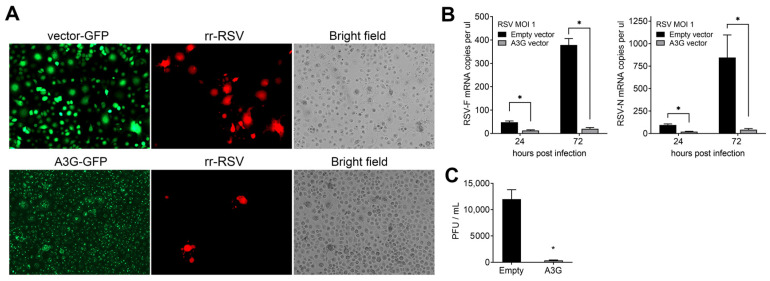
A3G is a host restriction factor against RSV. (**A**) Representative fluorescent and phase-contrast images (10×) of rr-RSV (RFP-tag) infection in H1299 cells stably expressing GFP alone (empty vector) or A3G-GFP tag infected for 72 h with RSV at MOI of 1. (**B**) RSV viral load measured by ddPCR for RSV-*F* and *-N* genes in H1299 cells overexpressing *A3G* and infected with RSV at MOI of 1. (**C**) RSV titer in H1299 as determined by plaque assay using supernatants of cells expressing empty vector or *A3G* and infected with RSV at MOI 1 for three days. * *p* < 0.05 of significance compared to empty vector.

After observing the high inducibility of p53-responsive A3G in A549 cells (Figure 1), we investigated its potential as a restriction factor against RSV. To this end, we established H1299 cell lines that stably overexpress GFP-tagged *A3G* (Figure 3), FLAG-tagged *A3G* (Figure S2A), and empty vector control cells. Despite *A3G* overexpression, the mRNA levels of other *A3* genes remained unchanged (Figure S3B). The cells were infected with rr-RSV, an RSV-A2 strain expressing recombinant RFP, at an MOI of 1 for 72 h. The *A3G* overexpression reduced the number of RFP-tagged RSV-infected cells and syncytial cells (Figure 3A). Similarly, H1299 cells overexpressing a FLAG-tagged *A3G* and infected with GFP-expressing RSV (rg-RSV) also had fewer syncytial cells (Figure S2C).

Furthermore, cells overexpressing *A3G* had fewer copies of the RSV-*F* and -*N* genes than those transfected with an empty vector and infected with the virus (Figure 3B). The *A3G*-overexpressing cells had lower G, N, P, and M viral protein products (Figure S2D) and decreased viral titers, as determined by plaque assays (Figure 3C). These results demonstrate that A3G can be a host restriction factor against RSV.

### 2.3. p53 Functional Status Influences RSV-Induced Cytopathic Effect (CPE) and Cell Death

**Figure 4:**
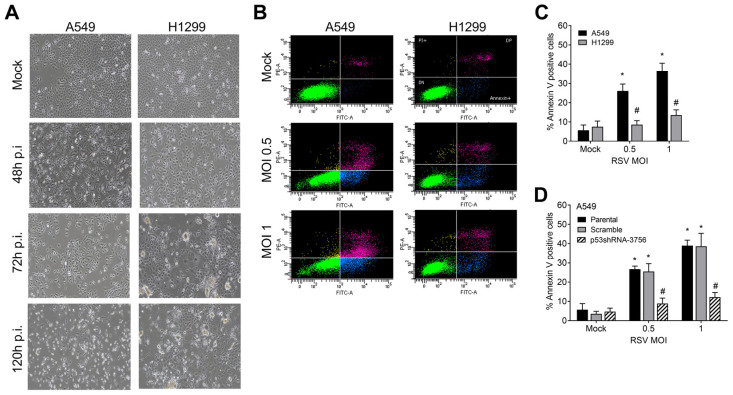
p53 functional status impacts CPE and apoptosis during RSV infection. (**A**) Representative microscopy (10×) bright-field images of time-course infection of RSV at MOI of 1 in A549 and H1299 cells. (**B**) Apoptosis assessment by Annexin-V/PI assay in A549 and H1299 cells infected with RSV for five days. The upper-right or lower-right quadrant mainly represents the necrotic/late-stage apoptotic cells or early stage of apoptotic cells, respectively, positive for annexin V binding and PI staining. Apoptosis quantification is presented in panel (**C**). * *p* < 0.05 of significance compared to mock condition; # *p* < 0.05 of significance compared to A549. (**D**) Reducing p53 expression by shRNAi in A549 cells reduces RSV-induced apoptosis. * *p* < 0.05 of significance compared to mock; # *p* < 0.05 of significance compared to scramble cells.

CPE changes that are characterized microscopically by the appearance of dead floating cells and syncytial cells were examined in RSV-infected A549 and H1299 cells. The A549 RSV-infected cells at an MOI of 1 had no noticeable CPE compared to the control mock-treated cells within the first 48 h of incubation (Figure 4A). However, by 120 h, there was a significant increase in floating dead cells and the appearance of few syncytial cells. Unlike A549 cells, the infection of p53-null H1299 cells led to a substantial increase in the appearance of syncytial cells and floating dead cells starting 72 h p.i. The differences in floating dead cells and syncytial cells between both cell lines were also clearly detected in RSV GFP-tagged infected cells at 72 h p.i. (Figure S3A). Reducing the p53 expression in p53shRNAi-A549 cells resulted in fewer floating dead cells and more appearances of syncytial cells at 120 h post RSV infection, suggesting a p53 role in preventing the formation of syncytial cells during RSV infection (Figure S3B).

These results led us to assess the viability of A549 and H1299 cells infected with RSV using an Annexin-V/PI flow cytometric assay that quantifies apoptotic cells (Figure 4B). At 120 h days p.i., the frequency of apoptotic A549 cells increased to ~26% and ~36% following infection with 0.5 and 1 MOI, respectively, compared to ~6% in the mock control group. Conversely, RSV infection had a small impact on the appearance of apoptotic cells in p53-null H1299 cells (Figure 4B,C). The reduction of p53 expression in A549 p53 shRNAi cells prevented RSV-induced apoptosis (Figure 4D). These findings show that p53 increases the expression of *A3* genes and impacts RSV-induced CPE and apoptosis. The possible relationship between p53-associated *A3* expression in RSV-infected cells and CPE or apoptosis remains to be established.

### 2.4. p53 Influences RSV-Induced Immune Responses at the Transcriptional Level

In recent years, a growing list of immune genes has been added to the vast number of genes directly targeted by p53 and the biological pathways that p53 monitors [12,14,35]. Using the A549/H1299 cell model system that we had previously employed in our p53/immune axis studies [17,35], we investigated the overall response of the p53/immune axis to RSV to understand its potential role in RSV challenges and the implications for other viral infections.

**Figure 5:**
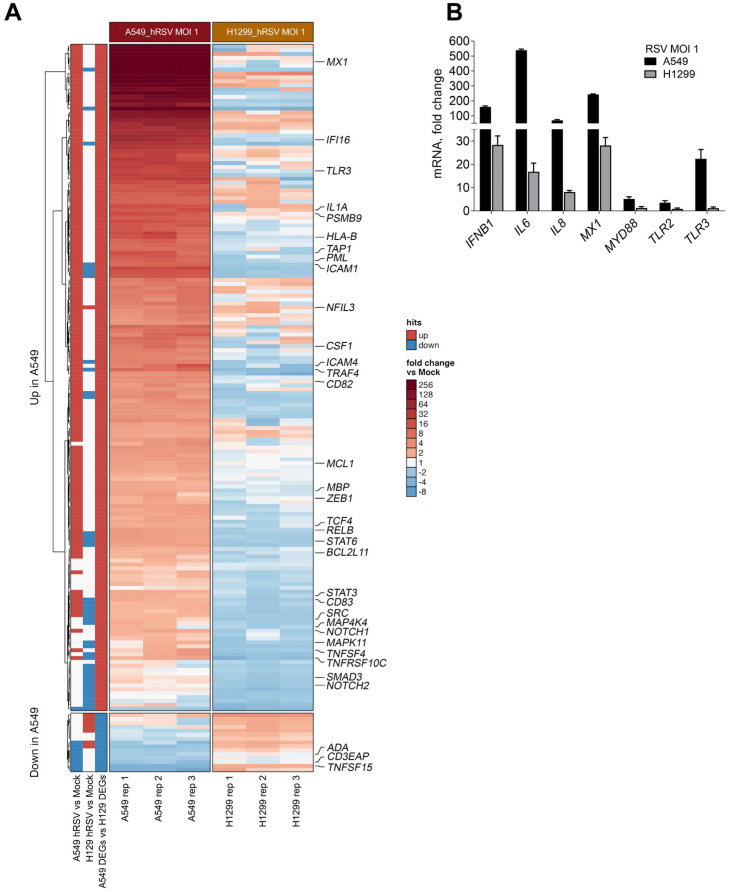
p53 influences RSV-induced immune response at the transcriptional level. (**A**) The expression of immunity-related genes using Nanostring nCounter technology in A549 and H1299 cells infected with RSV at MOI of 1 for three days. Shown are the fold-change induction values compared to the mock-infected condition. p53 known target cistrome genes are shown in large bold letters. (**B**) Validation of RSV-induced immune genes by qPCR in A549 and H1299 at 72 h p.i.

Using the Nanostring nCounter Immunology panel developed by NanoString technologies, the expression of 594 immune-related genes in both A549 and H1299 cells following RSV infection at an MOI of 1 at 72 h p.i. were analyzed. The immune panel did not include *A3* genes because the Nanostring screening system lacked discriminating probes for these genes. Differentially expressed genes (DEGs) were identified by fold-changes that were less or greater than 1.5 (*p* < 0.05; Table S2). RSV infection of A549 cells caused the altered expression of 37% (i.e., 217) of the 594 genes, while only 15% (i.e., 87) of the genes were altered following the H1299 infection relative to the mock uninfected cells. Among the DEGs in H1299, about 47 were also found among the A549 DEGs and only 12 had the same expression directionality (Table S2). Among the DEGs resulting from RSV infection in A549, most were due to upregulation (91%, 197/217). This contrasts with the H1299 DEGs, where 76% (66/87) were due to repression (Figure 5A). We validated the differential expressions in A549 and H1299 after RSV infection for the *IFNB1*, *IL6*, *IL8*, *MX1*, *MYD88*, *TLR2*, and *TLR3* genes (Figure 5B). In agreement with the enhanced p53-dependent apoptosis observed in RSV-infected A549 cells, we also found that several proapoptotic genes were significantly upregulated, including the genes coding for caspases 1, 2, 3, and 8, the death receptor *FAS*, *TNF*, *TNFGRSF10C*, *IFIT2*, and *IFI16* (see Table S2).

A total of 164 genes (153 upregulated and 11 downregulated) showed differential expression profiles between the A549 and H1299 cell lines after RSV infection (fold-changes of < or >1.5; *p* < 0.05; Table S3). We used the human p53 Binding And Expression Resource (BAER) to identify and link potential p53-binding peaks to p53-associated DEGs across the genome. The BAER was developed in our lab [35] from meta-analysis studies (41 published p53ChIPseq datasets) that involved a range of p53-activating agents (Doxorubicin, 5FU, IR, and Nutlin-3). With this tool, we found that 105 out of the 164 unique A549 DEGs had a p53-binding peak region in their transcriptional regulatory region in at least two independent studies. Additionally, 34 of the 105 hits had p53 binding and the associated gene expression (cistromes) change in the same study (indicated by large, bold gene lettering in Figure 5A).

As summarized in Table S3, there were ~70% (75/105) of the p53-linked genes that had p53-binding regions co-localized with regulatory elements (enhancers and promoters; USCS GeneHancer track tool [38]). This is consistent with our finding that p53 works as a potential transcriptional regulator for many RSV-related immune DEGs. Altogether, these findings suggest a broad role for p53 in response to RSV infection where, similar to the subset of *A3* genes, many of the RSV-related immune DEGs in human lung cells are potentially regulated directly by p53 at the transcriptional level.

## 3. Discussion

Host cell protein restriction factors can influence invasion by pathogens such as viruses during infection. RSV, like other viruses, is capable of evading these defenses [34]. In our study, we found that RSV-A2 strain infection causes the expression of several members of the *A3* gene family in different human primary and cancer lung cells, as well as colon cancer cell lines, suggesting a commonality of the RSV effect. A3 proteins are important for fighting viral infections, and we identified A3G as a new factor capable of restricting RSV infections. We also discovered that the master transcriptional regulator p53 plays a key role in activating *A3* genes in response to RSV infection, resulting in a 10- to 100-fold increase after RSV infection. Based on results with p53-proficient and -deficient cell lines, the p53-associated regulatory region occupancy, and increased *A3G* expression after RSV infection, we propose A3G is a p53-dependent restriction factor during RSV infection. Given their much lower levels of induction, it remains to be established if A3D and A3F can also be restriction factors.

As interferon-stimulated genes, *A3*s play a role in the innate immune response to and control of early virus infection. Activating these responses could increase A3 activity against many viruses [39]. Previously, we showed that p53 controls the expression of most *A3* genes in response to DNA damage and other cellular stresses, such as treatment with type I-IFN [16,17]. Those findings contrast with the p53-dependent late responses to RSV infection where the spectrum of expressed *A3* genes differed and p53-dependent upregulation was only observed for the *A3D*, *A3F*, and *A3G* genes. This is not surprising given the complex signaling responses to RSV cellular perturbation.

During RSV infection, *A3G* was found to be highly induced by p53. Furthermore, *A3G* overexpression reduced viral replication and viral-induced syncytia. A3 proteins might affect RSV replication via the deamination of cellular targets or viral genome sites or by direct interaction with viral RNA and proteins, thereby interfering with their functions. Although A3 proteins typically modify DNA templates, they can also edit RNA to defend against RNA viruses [40,41]. However, the cytidine deaminase function on RNA by A3 proteins has not been extensively characterized [42,43]. In the case of A3G, it binds with high affinity to ssRNA and ssDNA oligonucleotides in vitro [44,45], and the interaction with RNA induces the homo-oligomerization of A3G [45,46]. Nevertheless, many RNA viruses, including the hepatitis C virus, coronavirus HCoV-NL63, mumps virus, and measles virus, are restricted by A3 proteins lacking deaminase activity [22,47,48]. For the measles virus, which belongs to the same family as RSV, increased mutation frequencies were observed in the viral genome when A3G was overexpressed at physiological levels, independent of A3 deaminase activity [47]. Other studies have reported that A3G interacts with various proteins that regulate RNA metabolism and degradation and is part of ribonucleoprotein (RNP) complexes such as P bodies and stress granules in proliferating cells [42,49,50]. As proposed for other negative ssRNA viruses [51], it is possible that A3G-RSV RNA might be sequestered in P bodies for degradation. In addition, and as found with other viruses, some A3 proteins including A3G might interfere with viral replication through interaction with the viral machinery.

While the restriction mechanisms of p53-induced A3G remain to be determined, it would be interesting to assess its influence on infection by other viruses. For the case of A3D and A3F, both have been reported to modestly inhibit EV71 replication [52]. Milewaska and colleagues [22] reported the upregulation of *A3D*, *A3F*, and *A3G* transcripts in human airway epithelial cells infected with HCoV-NL63. In the same study, A3F limited the production of the progeny virus without causing hypermutation of the coronaviral genome. Future research should explore the roles of these proteins in RSV and other viral infections, especially given their family relationships, as well as p53-mediated gene expression.

Our research also shows that p53 activation by RSV influences the global cellular response against RSV and limits virus propagation by reducing RSV-induced CPE and triggering the apoptosis of infected cells. Reports of the relationship between RSV infection and p53 are limited and contradictory, largely due to the differences in experimental design, cellular types, and RSV strains used. In a study by Grosskreutz and colleagues [29], using human tracheobronchial epithelial cells, the RSV-A2 strain induced the downregulation of p53 during the early stages of infection (1 to 6 h) through the activation of its negative regulator Mdm2. This has a consequent impact on apoptosis. Interestingly, the authors observed that the effects of RSV infection are antagonized by Nutlin-3, a small specific molecule inhibitor that prevents the Mdm2/p53 association. Nutlin-3 treatment increased endogenous p53 expression in RSV-infected cells, causing earlier cell death. In another study, Machado et al. showed that the RSV-Long strain impaired p53 transcriptional activity at late stages of infection (48 h p.i.) in A549 cells, also via proteasome-dependent degradation [30]. Conversely, Eckardt-Michel and colleagues reported that the RSV-F protein expression in A549 cells caused the phosphorylation of p53 at serine 15 and the activation of p53 transcriptional activity, triggering the p53-dependent apoptosis of infected cells [28]. Moreover, Bian et al. observed that RSV-M expression also induced p53 and p21 accumulation in a time-dependent manner in A549 cells, reaching its maximal levels at 72 h p.i. [27].

Like other reports [27,29,30], we show that p53 protein levels in A549 cells do not increase in the early stages of infection (prior to 48 h). However, we observed that the activation of p53, as assessed by p53 Ser15 phosphorylation, occurs between 48 and 72 h after RSV infection. This activation leads to p53-related outcomes such as the transcriptional regulation of *A3* and other immune genes and apoptosis induction. The findings of Martinez et al. [53] support our results which indicate that RSV infection in A549 cells leads to DNA damage, causing p53 induction. Consistent with these observations, other studies have also shown that RSV infection activates the DNA damage response via ATM signaling [54] and induces oxidative stress through the increased production of reactive oxygen species (ROS) [55,56]; both of these are known activators of p53 [7,8].

In addition, p53 is also a type I IFN transcriptional target, indicating the importance of crosstalk between p53 and the IFN pathway in antiviral defense [15]. Furthermore, RSV infection can also activate p53 indirectly through other IFN-inducible proteins, such as protein kinase R (PKR), interferon-inducible protein IFIX, and promyelocytic leukemia protein (PML) [57,58,59]. We also found that p53 influences apoptosis and CPEs triggered by the RSV infection. Previous studies have reported that the RSV F protein activates p53 and induces caspase-dependent apoptosis [28]. Consistent with these observations, we also found that several apoptotic genes including *CASP1*, *CASP2*, *CASP3*, *CASP8,* and *FAS* were upregulated in A549 cells but not in p53-deficient H1299 cells. (We note that some genes commonly associated with apoptosis signaling were not included in the commercially available Nanostring code set used in our study.) Interestingly, we observed lung cancer cells infected with RSV produced more syncytia in cells lacking functional p53 (due to mutation or siRNA). This is likely due to the reduced ability to initiate apoptosis without p53. The possible relationship between the p53-associated *A3* expression in RSV-infected cells and CPE or p53-dependent apoptosis remains to be established.

In our study, we also addressed genome-wide p53-targeted immune gene responses to RSV infection using our model p53+ and p53- pair of lung cancer cell lines (A549 and H1299, respectively). Comparing both cell lines, we identified 164 DEGs after 72 h post RSV infection and found several potential direct p53 targets. The putative direct target DEGs were bound by p53 in other studies where p53 was activated pharmacologically or by stress, suggesting the involvement of p53 in regulating their expression. Our study specifically identifies 105 immune genes that can be added to our previously described p53/immune axis by virtue of their p53-dependent response to RSV infection. In support of the role of p53 as a transcriptional regulator during RSV infection, Xu and colleagues [50], using an ATAC-seq approach, found that, among 1120 genes in which RSV induced open chromatin domains, a significant fraction (~6.5%) of the genes have p53-binding motifs.

**Figure 6:**
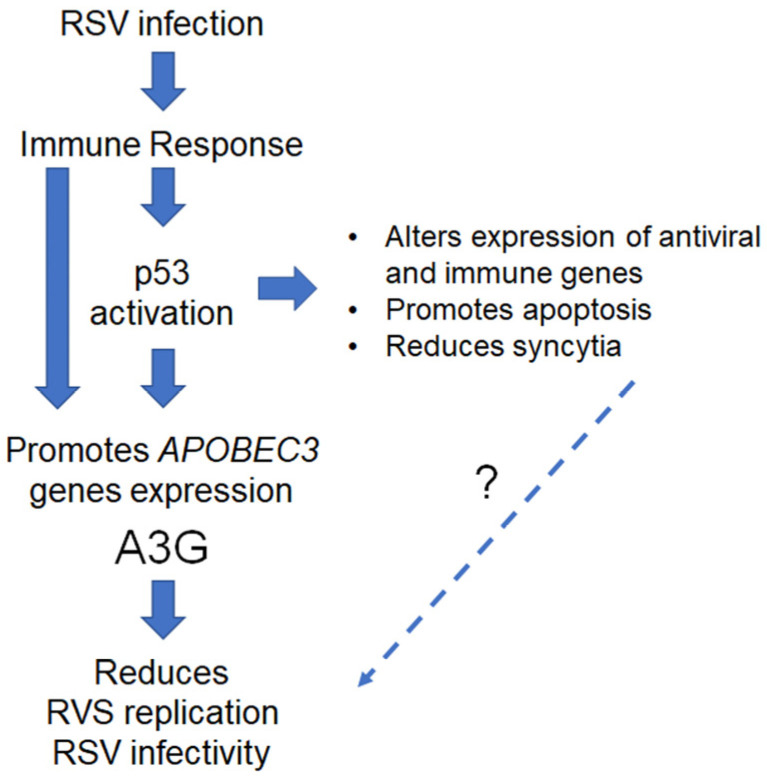
RSV infection activates the p53/immune axis transcriptional and apoptosis programs. RSV infection of lung epithelial cells leads to immune response signaling that involves the expression of several antiviral genes, including the *APOBEC3* genes, and the activation of p53. Activation of p53 also promotes the expression of various members of the *APOBEC3* gene family, mainly *A3G*. Overexpression of *A3G* in cells leads to reduced RSV replication and infectivity, although the exact mechanism is still unclear. Additionally, RSV-induced p53 activation results in the regulation of several other p53 transcriptional targets, such as apoptotic, antiviral, and immune genes. This, in turn, promotes the apoptosis of RSV-infected cells and reduces viral-induced syncytia. However, as denoted by the “?”, the impact of these additional p53-dependent activities in the course of RSV infection requires further study.

As summarized in Figure 6, our study on lung and cancer cell systems has demonstrated that p53 plays a crucial role in regulating various components of the immune response network following RSV infection. This finding suggests that the p53/immune axis pathways can be influenced by complex interactions, and our research shows that p53 can enhance and regulate immune responses post RSV infection. This is achieved by controlling immune genes and promoting crosstalk between biological pathways. When RSV infects the respiratory tract, the immune response is initiated by innate immune receptors on epithelial cells and alveolar macrophages. We identified several RSV immune pathway genes directly affected by p53’s presence. These include genes involved in IFN signaling pathways (*IFNB1*, *IFNAR1*, and *IFNAR2*) and TNF (*TNFSF4*, *TNFRSF10C*, and *TNFSF15*), innate immune receptors (*TLR2*, *TLR3*, and *MYD88*), and pro-inflammatory cytokine (*IL1A*, *IL6*, and *IL8*) genes. Several TLRs recognize RSV and signal via cytoplasmic adaptor proteins MyD88/TRIF to induce the expression of IFNs and other pro-inflammatory cytokines and chemokines such as *IL6* and *IL8*. In addition, IFNs bind to interferon-α/β receptors, causing the expression of hundreds of IFN-stimulated genes, including *TP53* gene [15], that can then amplify the antiviral responses and establish functional loops to boost the immune response against RSV.

Further complexity is exemplified by the relationship between the IFN, A3, and other p53 target genes. Due to IFN-elicited antiviral responses, *A3* gene expression can also be increased in response to various ligands of toll-like receptors [60]. Our previous studies have shown that p53 directly affects the expression of *TLR* genes in immune and cancer cell lines and enhances its downstream signaling [18,61]. For example, the induction of p53 directly increases *TLR3* expression, promoting apoptosis in the presence of TLR3 ligand poly I:C [62,63]. Interestingly, the activation of the TLR3 receptor by poly I:C boosts the expression of *A3G* and inhibits HIV replication [64]. These findings strongly suggest that the p53-dependent upregulation of *A3* and *TLR* expression can potentially contribute to enhanced innate immune responses during RSV infection.

Our findings confirm the significance of p53 in triggering a transcriptional boost for *A3G* and other immune genes that are involved in the host immune response against RSV and potentially other virus infections. It has been reported that other RNA viruses also activate p53. For example, the influenza virus (IAV), a member of the *Orthomyxoviridae* family of RNA viruses that causes human respiratory infections, leads to apoptosis mediated by the activation of p53, resulting in the reduction of IVA titers [65]. Surprisingly, it has been reported that the apoptosis of IAV-infected cells was required for the efficient propagation of IAV [66]. In comparison to the p53-WT mice, the p53-KO mice were more susceptible to IAV infection [67]. In another study, the upregulation of tested interferon-stimulated genes in p53-deficient cells was attenuated following exposure to IAV and interferon, suggesting that p53 plays an essential role in enhancing interferon signaling against IAV infection [68].

Moreover, the activation of p53 is an early and specific event during cell infection with Zika virus [69]. On the other hand, HIV-1 infection upregulates p53 in primary CD4+ T cells, leading to cell apoptosis through the activation of p53 target genes [70,71]. Increased p53-dependent apoptosis has also been observed in response to vesicular stomatitis virus (VSV), which was associated with reduced viral replication [15]. Recent studies on SARS-CoV-2 infection reveal a close association between COVID-19, p53 function, and signaling [72,73]. Importantly, RNA viruses have evolved mechanisms designed to abrogate p53 responses [11], suggesting that p53 has a broad role in antiviral defense. However, information on the consequences of the modulation of p53 activity in the context of several of these RNA viral infections is still limited.

Our study has several clinical implications. We have found that p53 can help regulate the immune response during RSV infection, indicating its potential as an immune adjuvant. Our previous research has shown that p53 can broadly control *TLR* genes, and we proposed that Nutlin-3, a p53 activator, could trigger *TLR* expression [13]. Clinical trials have examined Nutlin-3 and its derivatives [74,75], which can stabilize p53 and enhance its level even when activated by RSV infection. This would lead to increased amounts of p53-dependent gene expression. Clinically, the opportunity to boost p53 activity and levels of A3G might decrease the viral load. These approaches may also help in developing antiviral responses to other respiratory viruses such as influenza and coronaviruses. For instance, it was recently suggested that Nutlin-3, or its oral version Idasanutlin, could be beneficial in treating pulmonary infection induced by SARS-CoV-2 [76].

Finally, as noted above, we had previously shown that a difference in a TLR8 regulatory region affected its responsiveness to p53. This results in clinical outcome differences for RSV-infected children which led us to suggest treatment regimens that might be informed by the evaluation of TLR expression in response to p53 [31]. Having now defined a large p53/immune axis response to RSV in the present work, it would be interesting to identify SNPs that might affect the responsiveness of p53/immune axis genes and the possible impact on RSV infection.

## 4. Materials and Methods

### 4.1. Cell Lines

ATCC A549 (CCL-185), H1299 (CRL-5803), HEp2 (CCL-23), and MRC5 (CCL-171) cell lines were cultured as described by the vendor (ATCC). A549 cells stably expressing a scrambled or p53-directed shRNA were established, as previously described [16], with lentiviral scramble or p53-directed shRNA (p53shRNA-3756, TRCN0000003756, Sigma-Aldrich, St. Louis, MO, USA) and puromycin selection. Human colon cancer HCT116 p53−/− and p53+/+ cells were a gift from B. Vogelstein (John Hopkins University, Baltimore, MD, USA). Growth was described elsewhere [37]. All cells were cultured under standard conditions and regularly tested for mycoplasma (MycoAlert Mycoplasma Detection Kit, Lonza, Morrisville, NC, USA).

### 4.2. Viral Infections and Plaque Assays

Human RSV subtype A2 and its fluorescent GFP and RFP derivate strains were cultivated and titrated in HEp-2 cells. Dr. Mark Peeples (Rush-Presbyterian-St. Luke’s Medical Center, Chicago, IL, USA) provided rr-RSV and rg-RSV strains expressing red and green fluorescent protein genes. RSV infection occurred 4 h after cell plating in 6-well plates with an MOI of 0.5 and 1 in a low volume of serum-free medium. A complete medium was added 1 h post infection, and cells were incubated until harvesting at indicated times. For virus collection, cells were scraped, and the infectious media were pooled and centrifuged at 500× *g* for 5 min to remove cell debris. Non-infected monolayer cell supernatants were collected as noninfectious controls (mock, PBS 1×) for each respective virus. The RSV-induced cytopathic effects were evaluated by light and fluorescent microscopy (Zeiss automated inverted epifluorescent microscope, Zeiss, Oberkochen, Germany). Virus titers were determined by a plaque assay and were expressed as PFUs/mL. Briefly, Hep-2 cell monolayers in 6-well plates (24 h after plating) were adsorbed for one h with 10-fold dilutions of either RSV inoculum or cell culture supernatants. The infected monolayers were left for 6 ± 8 days under 1% methylcellulose (Sigma-Aldrich) overlay (in E-MEM with 2% FBS and antibiotic supplements) until plaques became visible. The cell monolayers were stained with 2% crystal violet in 10% ethanol for at least one hour. The stained monolayers were washed, air-dried, and clear plaques were counted manually under a light microscope. Virus titers were expressed as PFUs/mL.

### 4.3. RNA Isolation and qRT-PCR

Total RNA was isolated with an RNeasy kit (Qiagen, Germantown, MD, USA). Following the manufacturer’s recommendations, one µg total RNA was reverse-transcribed using an iScript cDNA synthesis kit (BioRad, Hercules, CA, USA). The *A3* gene expression was determined with standard procedures, primers, and Universal Primary Library System probes as described [77], while, for the remaining genes, TaqMan assay/probes were used (Table S4). Changes in mRNA levels were calculated with the 2^−ΔΔCt^ method using the expression from the housekeeping genes *TBP*, *B2M*, or *GUSB* for normalization.

### 4.4. Apoptosis Evaluation

Following the manufacturer’s instructions, the proportion of live and apoptotic cells after RSV infection was measured using an Annexin V-FITC/PI apoptosis detection kit (BD Biosciences, Franklin Lakes, NJ, USA). Live and apoptotic cells (Annexin-V-positive) were detected using a flow cytometer (LSR Fortessa and FACSDIva software version 9.0, BD Biosciences). Data were collected on 10,000 cells per condition and from at least three independent biological experiments.

### 4.5. Droplet Digital PCR (ddPCR)

Total RNA was isolated with Promega Maxwell RSC simplyRNA cell kits using a Promega Maxwell RSC 48 instrument following the manufacturer’s recommendations. Then, 500 ng total RNA was reverse-transcribed using an iScript cDNA synthesis kit (BioRad) following the manufacturer’s recommendations. The cDNA was diluted at 1:100 for the RSV genes. Changes in expression and copy number of genes of interest were quantified using the QX200 Droplet Digital PCR System, following the manufacturer’s instructions and under the following cycling protocol: 95 °C for 10 min followed by 40 cycles of 95 °C for 30 s and 55 °C for 1 min, followed by 98 °C for 10 min, followed by infinite 4 °C hold. The cycled plate was transferred and read in the FAM and HEX channels using the QX200 reader (Bio-Rad). Quanta-Soft software version 1.7 (Bio-Rad) estimated the number of template molecules per microliter of starting material.

### 4.6. Confocal Microscopy

Overnight cultures of A549 cells seeded at 3.5 × 10^5^ cells per well in 6-well plates containing 16 mm coverslips were infected with RSV at an MOI of 1. At 72 h after infection, the cells on coverslips were washed gently with PB. Samples were fixed with 4% paraformaldehyde (Sigma-Aldrich) in PBS for 10 min before permeabilization with 0.5% Triton X-100 (Sigma-Aldrich) for 5 min at room temperature. This step was followed by blocking with 5% bovine serum albumin in PBS-Tween 20 (0.1%) for 30 min at 37 °C. After washing with PBS twice, coverslips were immune-stained overnight with Alexa Fluor 647 conjugated monoclonal anti-p53 (PAb 1801) (ab193587, 1:100, Abcam, Boston, MA, USA) and FITC conjugated polyclonal anti-RSV (1:200, ab20391, Abcam) antibodies. After gentle washing with PBS, coverslip cells were stained for 10 min with 4′,6-diamidino-2-phenylindole (DAPI). This was followed by mounting with Prolong Gold (Thermo Fisher Scientifics, Durham, NC, USA) and examination on an LSM710 laser scanning confocal microscope (Zeiss, Oberkochen, Germany) using Zen Black Image software version 2.3 (Zeiss).

### 4.7. Western Blotting

Cell lysates were prepared by harvesting cells in RIPA lysis buffer (Thermo Fisher Scientifics) supplemented with halt protease inhibitors, PMSF, and sodium orthovanadate (Thermo Fisher Scientifics). After clearing the lysate by centrifugation, supernatants were collected, and total protein content was measured with BCA protein assay kit I (Thermo Fisher Scientific), according to the manufacturer’s instructions. Protein samples (10–30 μg) were resolved on 4–12% BisTris NuPAGE and transferred to polyvinylidene difluoride membranes (Thermo Fisher Scientific). After blocking with 5% non-fat dry milk, membranes were probed with the specific primary antibody followed by horseradish-peroxidase-conjugated goat anti-mouse or donkey anti-goat immunoglobulin (Santa Cruz Biotechnology, Santa Cruz, CA, USA) and developed using SuperSignal West Femto Maximum Sensitivity Substrate (Cat# 34095, Thermo Fisher Scientific). The following are the primary antibodies used: p53 (DO-1), Actin (C-11), Lamin B1 (G-1) from Santa Cruz Biotechnology, RSV (ab20745 Abcam), anti-DDK (FLAG, Clone OTI4C5, Origene, Rockville, MD, USA), and turboGFP (OTI2H8, Origene).

### 4.8. Plasmid Transfection

Expression vectors pCMV6-AC-GFP, pCMV6-AC-Myc-DDK, pCMV6-AC-APOBEC3G-GFP-tagged, and pCMV6-AC-APOBEC3G-Myc-DDK-tagged were obtained from OriGene. Transient transfections were carried out using FuGENE 6 reagent (Promega, Madison, WI, USA), according to manufacturer’s instructions.

### 4.9. Chromatin Immunoprecipitation (ChIP)

ChIP assays were performed as described previously [16]. The chromatin was sheared by sonication (three 15 min cycles of 30 s on and 30 s off) using a Bioruptor device (Diagenode, Denville, NJ, USA). DNA was isolated after immunoprecipitation with either a mouse IgG (negative control) or p53 antibody DO-1 (Santa Cruz Biotechnology). Real-time PCR and melting curve analysis were performed in triplicate using the SYBR^®^ Green (Applied Biosystems, Foster City, CA, USA) dye detection method on the ABI PRISM 7900 HT Sequence Detection System under default conditions. Primers are described in Supplemental Table S1. The comparative Ct method was used for the quantification. The enrichment of specific targets was calculated as the fraction of Input (%) of DNA area of interest recovery in p53-immunoprecipitated or nonspecific IgG control samples.

### 4.10. NanoString nCounter Gene Expression

The nCounter Immunology platform (NanoString Technologies, Seattle, WA, USA) was used for mRNA detection of a 594-gene panel, according to manufacturer’s instructions [78]. This custom panel is a collection of immunity-related genes. Briefly, 300 ng of total RNA were hybridized to the probes at 67 °C for 18 h using a thermocycler. After loading into the nCounter Prep Station (NanoString Technologies) for purification and immobilization, the sample cartridge was transferred and imaged on the nCounter Digital Analyzer (NanoString Technologies). Data were analyzed in R version 3.6.1. Log2 expression values were normalized using the geometric mean of the 15 NanoString nSolver housekeeper genes (*ABCF1*, *ALAS1*, *EEF1G*, *G6PD*, *GAPDH*, *GUSB*, *HPRT1*, *OAZ1*, *POLR1B*, *POLR2A*, *PPIA*, *RPL19*, *SDHA*, *TBP*, and *TUBB*). Data QC was performed using MA-plots with R package jamma [79] to confirm low variability for all genes within sample groups and low variability for housekeeper genes across sample groups. No sample outliers were observed. NanoString negative controls and MA plots were used to define a threshold of 32 counts for statistical filtering. Comparisons were performed using limma-voom [80]. Dysregulated genes were required to have a minimum expression of 32 normalized counts in at least one sample group, a fold-change of 1.5 or higher, and a Benjamini–Hochberg adjusted *p*-value of 0.01 or lower. Heatmaps were prepared with ComplexHeatmap version 2.7.8.1000 [81].

### 4.11. Statistical Analysis

All data are presented as the mean ± SD from at least three separate experiments. Statistical analysis was performed using GraphPad Prism, version 9 (GraphPad Software) with the following tests: assess differences between study groups: U-Mann–Whitney and one-way ANOVA. The level of statistical significance was taken at *p* < 0.05.

## 5. Conclusions

Our study found that RSV infection activates p53, leading to the altered p53-dependent expression of three out of seven of the *A3* genes *A3D*, *A3F*, and *A3G*, along with p53 site-specific binding. *A3G* overexpression can effectively reduce RSV viral replication and syncytial induction. Furthermore, nearly 100 genes can be directly targeted by the p53/immune axis during RSV infection. The findings have significant implications for RSV clinical and therapeutic studies, including using p53 adjuvants to boost the response of *A3* genes.

## Data Availability

All data generated during this study are included in this article. The p53 Binding And Expression Resource (BAER) can be accessed here: https://www.niehs.nih.gov/research/resources/databases/p53/index.cfm. Accessed on 10 October 2023.

## Supplementary Materials

The following supporting information can be downloaded at: https://www.mdpi.com/article/10.3390/ijms242316793/s1.
