# Supplementary material for: APOBEC3G Is a p53-Dependent Restriction Factor in Respiratory Syncytial Virus Infection of Human Cells Included in the p53/Immune Axis
Source: Int J Mol Sci. 2023 Nov 27;24(23):16793. doi: 10.3390/ijms242316793 (PMC10706465; doi:10.3390/ijms242316793)
Supplement: Supplementary file 1 [file ijms-24-16793-s001.zip › IJMS_Gladwell_Supplementary material_acepted version.pdf]

Figure S1

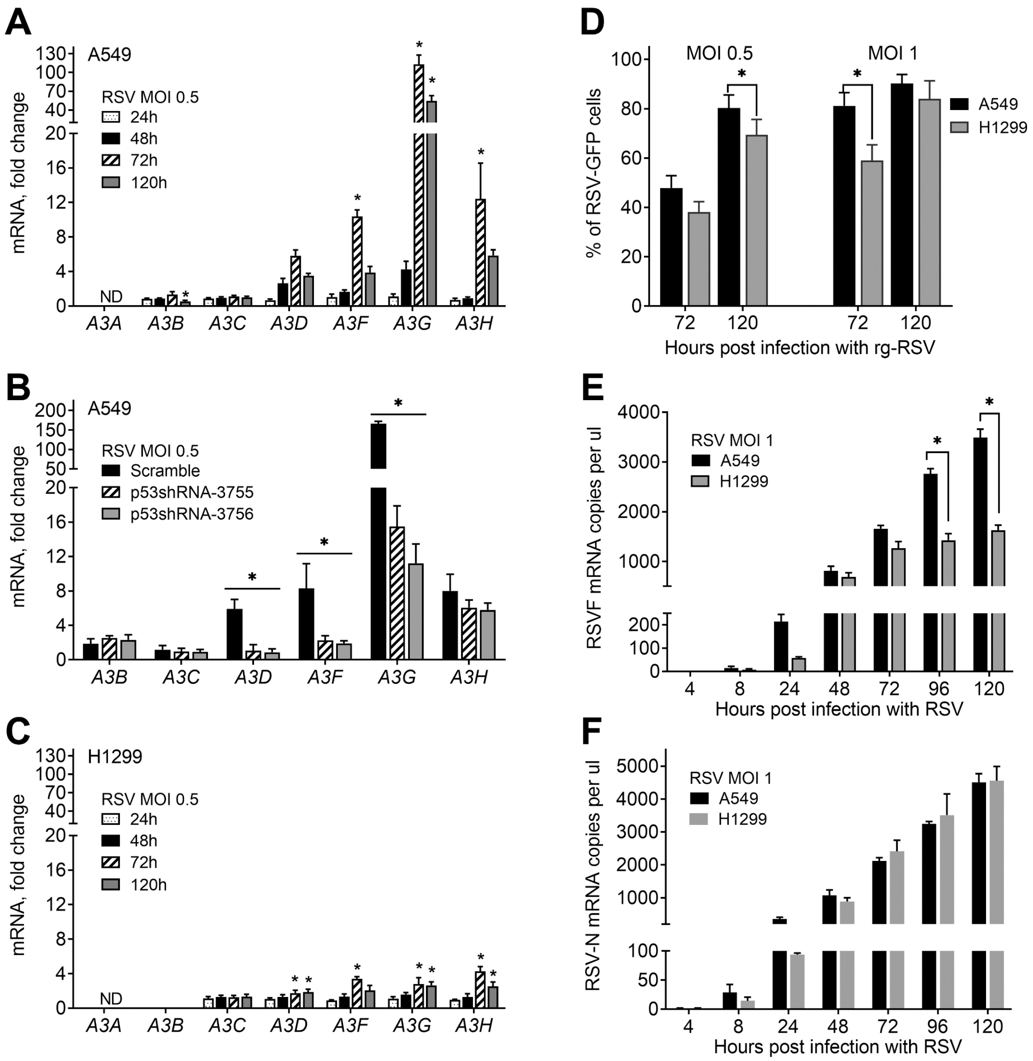

Supplementary Figure 1. RSV infection-induced expression of *A3* genes in human lung cells. (**A**) Time course of induced expression of *A3* genes in A549 cells infected with RSV at MOI of 0.5 determined by qPCR. Expression changes are presented as fold-change relative to mock (PBS) non-infected cells. *, P < 0.05 compared with non-infected cells. (**B**) Expression of *A3* genes in A549 cells stably expressing scrambled shRNA or p53 shRNAi (p53sh-3755 or p53sh-3756) vectors and infected for 72 h with RSV at MOI of 0.5. Values are displayed as fold-changes relative to their respective parental A549 cells. *, P < 0.05 compared to scramble cells. (**C**) Time course of induced expression of *A3* genes in H1299 cells infected with RSV at MOI of 0.5. (**D**) Time course and dose-response infection of rg-RSV (GFP- tag) in A549 and H1299 cells. At the indicated times, green RSV infected cells were counted by flow cytometry. Time course of RSV viral load measured by ddPCR for (**E**) RSV-*F* and (**F**) RSV-*N* genes in A549 and H1299 cells infected with RSV-A2 at MOI of 1. *, P < 0.05 compared with A549 cells.

Figure S2

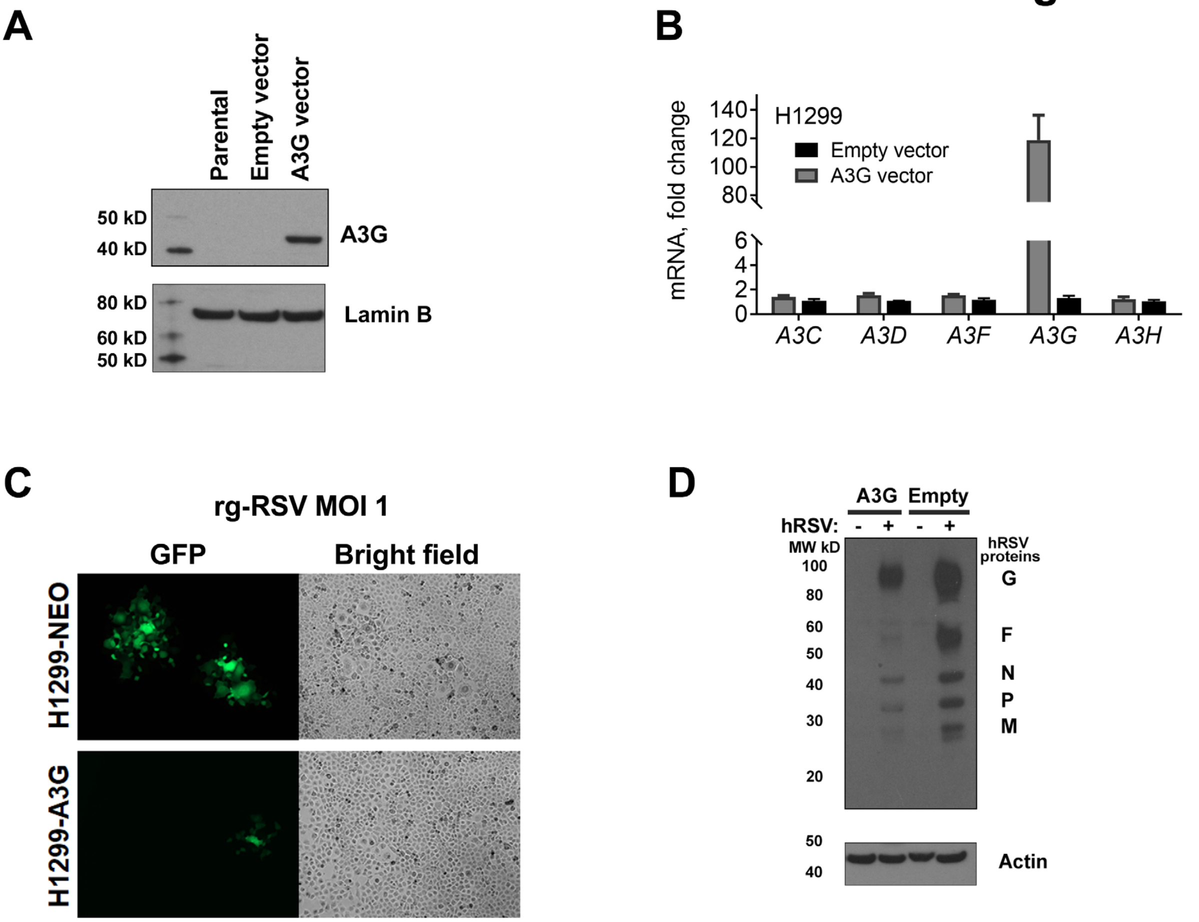

Supplemental Figure 2. Overexpression of *A3G* reduces RSV infection. (**A**) Representative immunoblot of H1299 cells overexpressing an A3G-Flag tag expression vector. (**B**) *A3* gene family expression profile in A3G-H1299 cells determined by qPCR. (**C**) Representative fluorescent and phase-contrast images of empty vector and A3G-FLAG tag H1299 cells infected for 48 h with rg-RSV (GFP tag) at an MOI of 0.5 (10×). (**D**) Comparison of RSV protein levels in H1299 cells transfected with empty vector or A3G-GFP tag vector and infected with rr-RSV (RFP-tag) during 72 h at MOI of 1.

Figure S3

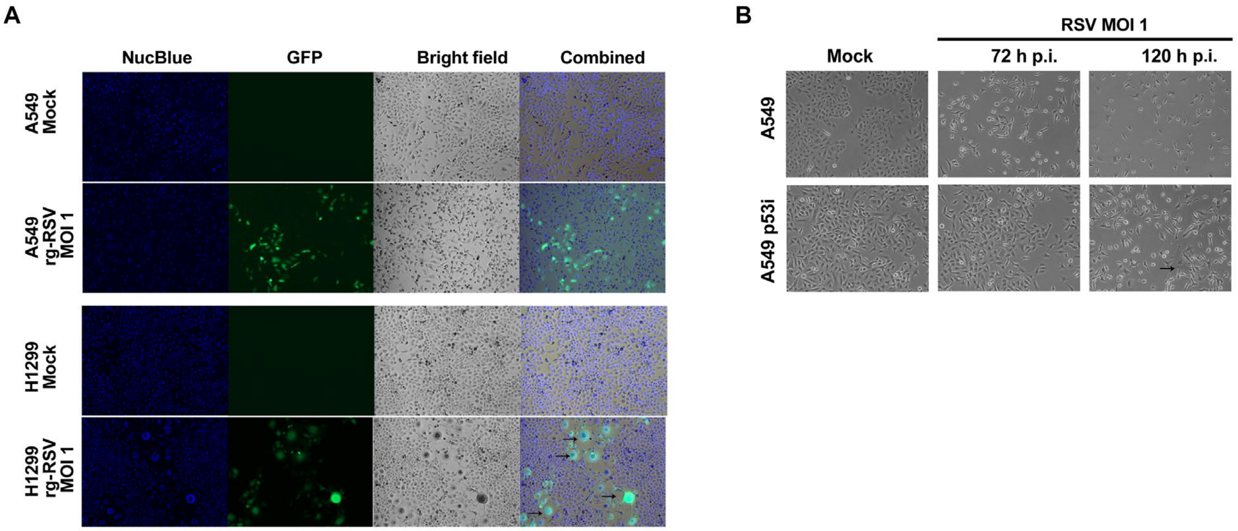

Supplemental Figure 3. p53 functional status impacts RSV-induced syncytia formation and cell death. (**A**) Fluorescent and phase-contrast images of A549 and H1299 cells infected with rg-RSV (GFP tag) strain at MOI 1 for three days (10×). NuncBlue stain was added to the cell cultures before its visualization under the microscope to label cell nuclei. (**B**) Bright-field microscope images of RSV infected parental A549 and p53shRNAi (10×) cells. The cells were infected with MOI of 1. The black arrows indicate syncytial cells.

Table S1. qPCR Ct values of APOBEC3 genes in human cell lines.

	A549		H1299		MRC5		HCT116 p53+		HCT116 p53-	
Gene	Ct	Stdv	Ct	Stdv	Ct	Stdv	Ct	Stdv	Ct	Stdv
APOBEC3A	ND		ND		ND		ND		ND	
APOBEC3B	35.6	0.670	ND		27.2	0.356	24.9	0.427	25.3	0.469
APOBEC3C	25.9	0.686	26.5	0.587	23.2	0.439	25.1	0.242	25.4	0.328
APOBEC3D	32.4	0.812	26.7	0.626	31.9	0.495	33.5	0.534	34.2	0.363
APOBEC3F	28.0	0.796	27.7	0.741	30.1	0.553	29.1	0.138	29.4	0.177
APOBEC3G	36.6	0.762	31.5	0.844	27.7	0.408	27.9	0.643	29.8	0.588
APOBEC3H	31.9	0.659	34.2	0.642	31.1	0.319	29.6	0.632	29.2	0.355

Note: Universal Primary Library System (Roche) was used for A3 expression.
The total cycles for the qPCR reaction was set to 45 as recommended by manufacturer

Table S2: Differentially expressed genes in A549 and H1299 cells infected with RSV MOI 1, 3 days.

See attached file

Table S3: P53 Cistrome genes for RSV differential expressed genes.

See attached file

Table S4. List of primers and probes and gene expression assays.

Gene	Primer / Probe	Seq (5'->3')	Assay	UPL probe	Vendor
APOBEC3A	A3A_2742	GAGAAGGGACAAGCACATGG	qPCR	UPL26	Roche
	A3A_2743	TGGATCCATCAAGTGTCTGG	qPCR		
APOBEC3B	A3B_3220	GACCCTTTGGTCCTTCGAC	qPCR	UPL1	Roche
	A3B_3221	GCACAGCCCCAGGAGAAG	qPCR		
APOBEC3C	A3C_3085	AGCGCTTCAGAAAAGAGTGG	qPCR	UPL155	Roche
	A3C_3086	AAGTTTCGTTCCGATCGTTG	qPCR		
APOBEC3D	A3_D2749	ACCCAAACGTCAGTCGAATC	qPCR	UPL51	Roche
	A3D_2750	CACATTTCTGCGTGGTTCTC	qPCR		
APOBEC3F	A3F_2751	CCGTTTGGACGCAAAGAT	qPCR	UPL27	Roche
	A3F_2752	CCAGGTGATCTGGAAACACTT	qPCR		
APOBEC3G	A3G_2753	CCGAGGACCCGAAGGTTAC	qPCR	UPL79	Roche
	A3G_2754	TCCAACAGTGCTGAAATTCG	qPCR		
APOBEC3H	A3H_2757	AGCTGTGGCCAGAAGCAC	qPCR	UPL21	Roche
	A3H_2758	CGGAATGTTTCGGCTGTT	qPCR		
TBP	TBP_3231	CCCATGACTCCCATGACC	qPCR	UPL51	Roche
	TBP_3232	TTTACAACCAAGATTCACTGTGG	qPCR		
IFNB1	Hs01077958_s1		qPCR		Applied Biosystems Thermo Fisher Scientific
IL6	Hs00174131_m1		qPCR		Applied Biosystems Thermo Fisher Scientific
IL8	Hs00174103_m1		qPCR		Applied Biosystems Thermo Fisher Scientific
MX1	Hs00895608_m1		qPCR		Applied Biosystems Thermo Fisher Scientific
MYD88	Hs00182082_m1		qPCR		Applied Biosystems Thermo Fisher Scientific
TLR2	Hs00152932_m1		qPCR		Applied Biosystems Thermo Fisher Scientific
TLR3	Hs00152933_m1		qPCR		Applied Biosystems Thermo Fisher Scientific
B2M	Hs00187842_m1		qPCR		Applied Biosystems Thermo Fisher Scientific
GUSB	Hs00939627_m1		qPCR		Applied Biosystems Thermo Fisher Scientific
APOBEC3C	A3C p53RE +80_F	CCAGTCCGCCTGCTGAGA	ChiP-PCR		Integrated DNA Technologies
	A3C p53RE +80_R	GCTCAAATCATCCTTTGGTTCAA	ChiP-PCR		Integrated DNA Technologies
APOBEC3F	A3F p53RE+717_ F	CCGCCACCGAAAGTCATG	ChiP-PCR		Integrated DNA Technologies
	A3F p53RE+717_ R	TCAAAGCACTGTGAACAAAATTCC	ChiP-PCR		Integrated DNA Technologies
APOBEC3G	A3G p53RE +722_F	GGACTGGGAAGGCCTAGAAGA	ChiP-PCR		Integrated DNA Technologies
	A3G p53RE +722_R	AAATGGCCCTGCAAAGTTGT	ChiP-PCR		Integrated DNA Technologies
APOBEC3H	A3H p53RE -67_F	CACTCCAGTCCCACAAAAGGA	ChiP-PCR		Integrated DNA Technologies
	A3H p53RE -67_R	GGCGGCAGTACCTGATCTGT	ChiP-PCR		Integrated DNA Technologies
RSV F	Forward	GCAATTACCACAATCCTCAC	ddPCR		BioRad
	Reverse	GAGCACTAAGATAGCCTTTG	ddPCR		
	Probe	FAM-ATCAATCAACATGCAGTGCAGTTAG	ddPCR		BioRad
RSV G	Forward	AGATCAACTTCTGTCATCCAGCAA	ddPCR		BioRad
	Reverse	TTCTGCACATCATAATTAGGAGTAT	ddPCR		
	Probe	HEX-ATACACCATCCAACGGAGCACAGG	ddPCR		BioRad
